# Parasitic Nematodes Modulate PIN-Mediated Auxin Transport to Facilitate Infection

**DOI:** 10.1371/journal.ppat.1000266

**Published:** 2009-01-16

**Authors:** Wim Grunewald, Bernard Cannoot, Jiří Friml, Godelieve Gheysen

**Affiliations:** 1 Department of Plant Systems Biology, Ghent University, Ghent, Belgium; 2 Department of Molecular Biotechnology, Ghent University, Ghent, Belgium; The University of North Carolina at Chapel Hill, United States of America

## Abstract

Plant-parasitic nematodes are destructive plant pathogens that cause significant yield losses. They induce highly specialized feeding sites (NFS) in infected plant roots from which they withdraw nutrients. In order to establish these NFS, it is thought that the nematodes manipulate the molecular and physiological pathways of their hosts. Evidence is accumulating that the plant signalling molecule auxin is involved in the initiation and development of the feeding sites of sedentary plant-parasitic nematodes. Intercellular transport of auxin is essential for various aspects of plant growth and development. Here, we analysed the spatial and temporal expression of PIN auxin transporters during the early events of NFS establishment using promoter-GUS/GFP fusion lines. Additionally, single and double *pin* mutants were used in infection studies to analyse the role of the different PIN proteins during cyst nematode infection. Based on our results, we postulate a model in which PIN1-mediated auxin transport is needed to deliver auxin to the initial syncytial cell, whereas PIN3 and PIN4 distribute the accumulated auxin laterally and are involved in the radial expansion of the NFS. Our data demonstrate that cyst nematodes are able to hijack the auxin distribution network in order to facilitate the infection process.

## Author Summary

Sedentary plant-parasitic nematodes are major agricultural pests worldwide. They establish an intimate relationship with their host and force the latter to induce specialized structures from which they feed. Due to the withdrawal of nutrients together with the damage they cause to the hosts roots, plant-parasitic nematodes are responsible for global agricultural losses amounting to an estimated $157 billion annually. Understanding the plant-nematode interaction at the molecular level can create novel approaches to control plant-parasitic nematodes. Fascinatingly, nematodes are able to manipulate host developmental pathways to their own advantage. Here, we focus on the plant signalling molecule auxin. Intercellular transport of auxin is essential for various aspects of plant growth and development and it was shown that it accumulates during the early stages of nematode infection. We studied the expression and the role of PIN auxin transporters during nematode infection and could show that nematodes manipulate the auxin distribution networks in order to induce their feeding sites. Our results explain for the first time the mechanism behind the dynamic auxin gradients during NFS development.

## Introduction

Plant-parasitic nematodes are major agricultural pests world-wide and are responsible for global agricultural losses amounting to an estimated $157 billion annually [1]. Most of the damage is causes by sedentary plant-parasitic nematodes of the family *Heteroderidae* which transform differentiated plant root cells into nematode feeding sites (NFS). Cyst nematodes such as *Heterodera schachtii* induce syncytia by cell wall dissolution and subsequent fusion of the infected cell with its neighbouring cells. Root-knot nematodes such as *Meloidogyne* spp. induced five to seven multinucleated giant cells by repeated cycles of mitosis without cytokinesis [2]. It has been proposed that plant-parasitic nematodes manipulate the developmental programs of their hosts to induce the NFS [3]. Plant development and growth is a tightly regulated process in which phytohormones play crucial roles. The idea that auxin, the rooting hormone par excellence, could be involved in the nematode infection process was launched as early as the sixties. Balasubramaniam and Rangaswami [4] were the first to identify indole compounds, the precursors of auxins, in extracts from *Meloidogyne javanica* infected tomato roots. Several studies have illustrated this reliably over the years in different nematode-host interactions [5]–[7]. Since then an increasing amount of data points towards an important role for auxin in NFS establishment. In 1971, Kochba and Samish [8] showed that application of auxin (NAA) to resistant peach made them susceptible to *M. javanica* and Glazer *et al.* [9] reported that application of IAA to tomato roots infected with *M. javanica* resulted in a concentration-dependent increase of gall fresh weight. On the other hand, the auxin-insensitive tomato mutant *dgt* was shown to be *de facto* resistant to cyst nematodes, while in the model plant *Arabidopsis thaliana* a significant reduction in the number of developing cyst nematodes was observed in the *axr2/iaa7* mutant that is defective in auxin signalling [10]. More recently in *Arabidopsis*, the upregulation of the auxin responsive DR5 reporter could be demonstrated shortly after nematode infection [11],[12]. This local auxin accumulation could be due to auxin directly secreted by the nematode [13] or could be a result of directional auxin transport towards the feeding site initial. Auxin, mainly produced by the aerial parts of the plant is basipetally transported to the root tip by means of a complex interacting network of influx and efflux systems. Briefly, transmembrane proteins of the AUX1/LAX family, part of the influx system [14]–[17] and the PIN family, mediating auxin efflux [18], guide the direction of the auxin flow on account of their subcellular polar localisations. The polar localisation of PIN proteins can be modulated by developmental [19] and external cues [20] thereby being of major importance in creating asymmetric auxin distribution patterns or so-called auxin gradients. Since it has been shown that these transport-dependent auxin gradients represent a common module for the formation of all plant organs [21], it is not unlikely that also the increased DR5 activity accompanying nematode infection is the result of PIN-mediated auxin transport. In support of this, infection studies using mutants defective in PIN1 and PIN2 have revealed a significant reduction in development of the cyst nematode *Heterodera schachtii* [10]. Moreover application of the synthetic auxin transport inhibitor NPA hampers the expansion of syncytia resulting in a reduction of nematode development [10].

Here, we investigate the collective contribution of PIN-dependent auxin distribution networks in early NFS formation. In situ expression studies using promoter-*GUS/GFP* fusions revealed temporal and spatial expression patterns of *PIN1*, *PIN2*, *PIN3*, *PIN4* and *PIN7* during the early events of NFS establishment. Additionally we infected single and double *pin* mutants to analyse their role during the nematode infection process. Our results clearly demonstrate that the plant-parasitic nematode *H. schachtii* manipulates its host auxin distribution network in order to induce NFS and explain for the first time the mechanism behind the dynamic auxin gradients during NFS development.

## Results

### Auxin Response and *PIN* Gene Expression during Early Nematode Feeding Site Establishment

**Figure 1:**
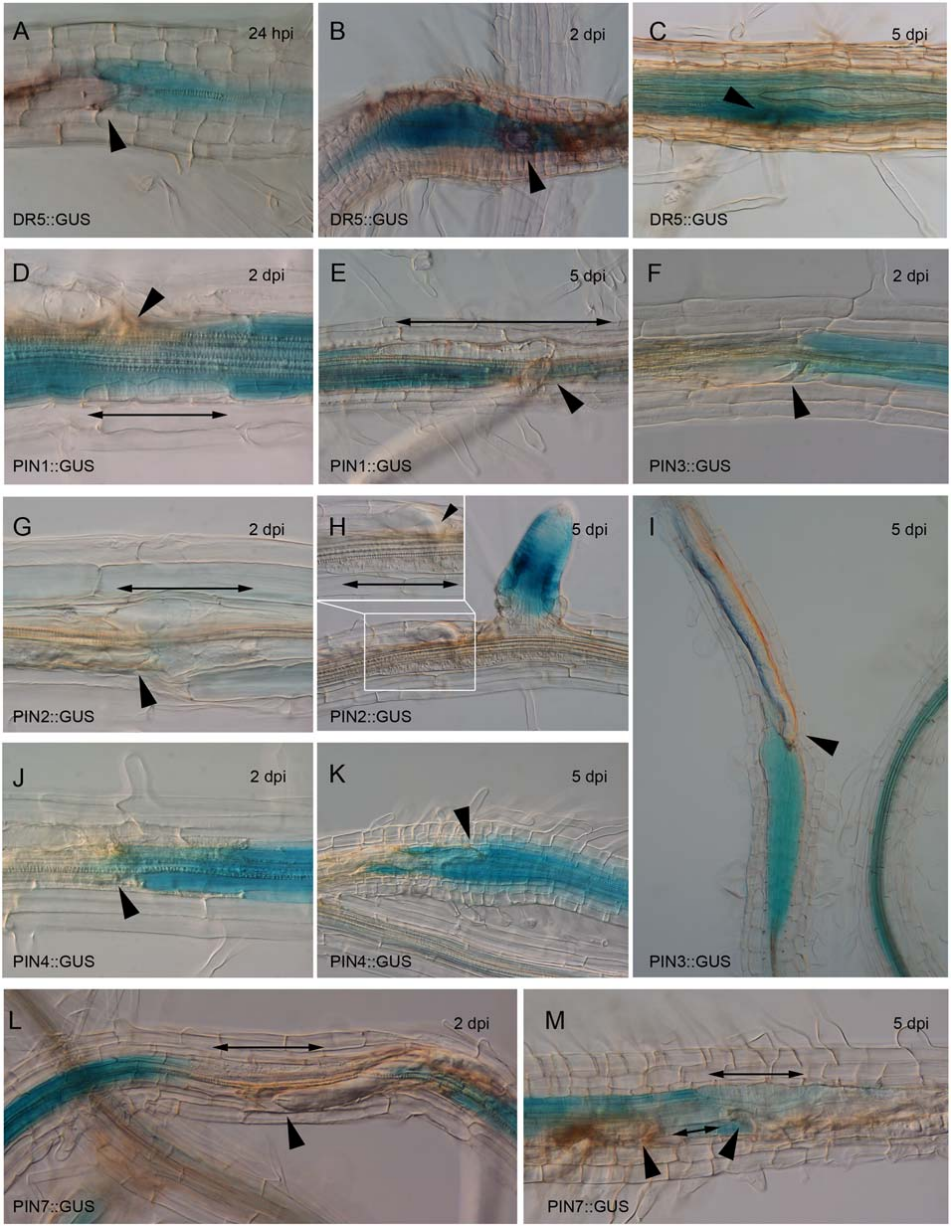
Expression of *PIN* genes upon infection with *H. schachtii*. (A–C) DR5 activity in NFS at 24 hpi (A), 2 dpi (B) and 5 dpi (C). NFS is outlined in (C). (D,E) *PIN1::GUS* expression is absent in NFS at 2 dpi (D) and 5 dpi (E). (G,H) *PIN2* is not expressed at 2 dpi (G) nor at 5 dpi (H). Inset is close-up of nematode head and feeding site. (F,I) *PIN3* is highly expressed at 2 dpi (F) and 5 dpi (I). (J,K) *PIN4::GUS* expression at 2 dpi (J) and 5 dpi (K). (L,M) *PIN7* is not expressed in NFS at 2 dpi (L) nor at 5 dpi (M). Single arrowheads point to nematode heads; double headed arrows indicate NFS when not GUS-stained.

In Arabidopsis roots, auxin accumulation can be easily visualized at the cellular level with the auxin responsive reporter *DR5::GUS* [22]. As reported earlier, DR5 activity can be detected from the very early events of nematode feeding site (NFS) establishment onwards (Figure 1A) [11],[12]. The GUS staining persists in the developing syncytium until 2 days post inoculation (dpi) (Figure 1B). Conceptually, the enhanced auxin response in NFS can be accomplished by auxin secreted by the nematode, local biosynthesis of auxin or by local changes of the host's auxin transport. Later on during nematode infection, the GUS staining becomes less specific to the syncytium and is more pronounced in the periphery of the NFS (Figure 1C). This suggests a role for auxin in the preconditioning of cells prior to their integration into the developing syncytium and implies that nematodes can change the flow and accumulation of auxin along the root.

The best characterized components of auxin transport are the PIN efflux facilitators. The *PIN* genes described so far are expressed in specific but overlapping regions of the root (Figure S1) [23]. To examine which PIN proteins might play a role in auxin distribution during NFS formation, we analyzed *PIN* expression during early nematode infection (2 and 5 dpi) using *PIN1*, *2*, *3*, *4*, and *PIN7::GUS* transgenic plants. These *PIN::GUS* fusions have been characterised in detail previously and shown to display in the root the same expression pattern as endogenous proteins [19],[20],[24],[25]. At 2 dpi both *PIN3* and *PIN4* were highly and very specific expressed in the young syncytia of *H. schachtii* (Figure 1F and 1J). This expression was maintained at 5 dpi (Figure 1I and 1K) and implicates an important role for PIN3 and PIN4 in NFS development. Interestingly a complete opposite expression behaviour was observed for *PIN1* and *PIN7*. While both genes are expressed in the vasculature of infected and uninfected roots, no GUS staining could be detected in the feeding structures (Figure 1D–1E, 1L–1M). This suggests an active downregulation of both genes by the infecting nematodes. Finally, no *PIN2::GUS* activity could be observed in NFS at 2 nor at 5 dpi (Figure 1G and 1H).

These results demonstrate that parasitic nematodes exhibit a complex modulation of the PIN-dependent auxin distribution network involving both the upregulation and downregulation of *PIN* expression.

### Nematodes Manipulate PIN Polarity in NFS

**Figure 2:**
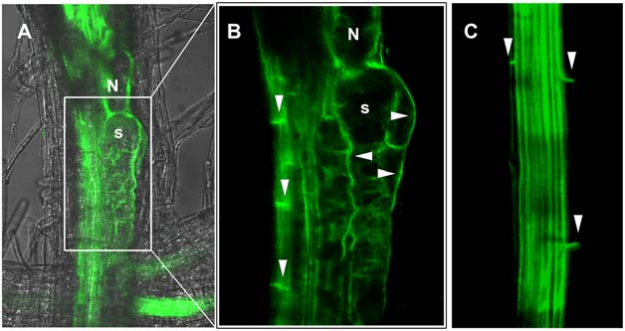
PIN3-GFP localisation upon infection with *H. schachtii*. (A) Merged GFP and transmission confocal image of a syncytium at 4 dpi. (B) Detail of (A) showing basal localisation in vascular tissue and lateral localisation in the syncytium. (C) Uninfected PIN3::PIN3-GFP control root showing basal PIN3-GFP localisation of pericycle cells. N, nematode; s, syncytium; arrows show asymmetric PIN localisation.

In order to confirm the expression behaviour of the *PIN* genes during nematode infection and to address whether nematodes can manipulate PIN polarity, PIN translational fusion lines driven by their endogenous promoters (*PIN::PIN-GFP*) were analyzed upon infection with *H. schachtii*. Confirming the above-mentioned results, only *PIN3* and *PIN4* showed a strong expression in NFS (Figure S2). In the vasculature of uninfected plants, PIN3 proteins are localized at the basal side of the cell membrane as well as at the inner membranes of pericycle cells (Figure 2C) [20]. However, the polarity of PIN proteins can be rapidly modulated in order to react to developmental or external cues [19],[20]. Interestingly, in contrast to the basal GFP signal in the vasculature, clear fluorescence could be detected at the outer and inner lateral sides of 4-days old syncytia (Figure 2A and 2B). The same could be observed in NFS induced in *PIN4::PIN4-GFP* roots (Figure S2). To ensure that PIN-GFP relocation does not arise as a consequence of the altered morphology of the infected cells, we performed a time-course experiment from 12 hours post inoculation until 2 dpi. The same lateral GFP signal as was observed in 4-days old syncytia could be seen in very young feeding sites (Figure S3). This shows that nematodes can indeed change PIN polarity to fine-tune auxin fluxes at the site of infection. Furthermore, this result correlates with the observed change in DR5 staining pattern at the feeding site and strongly suggests that PIN3 and PIN4 orchestrate auxin transport to the cells surrounding the NFS so that these cells can be primed for integration in the expanding NFS.

### Disturbing PIN-Mediated Auxin Transport Affects NFS Initiation and Development

**Figure 3:**
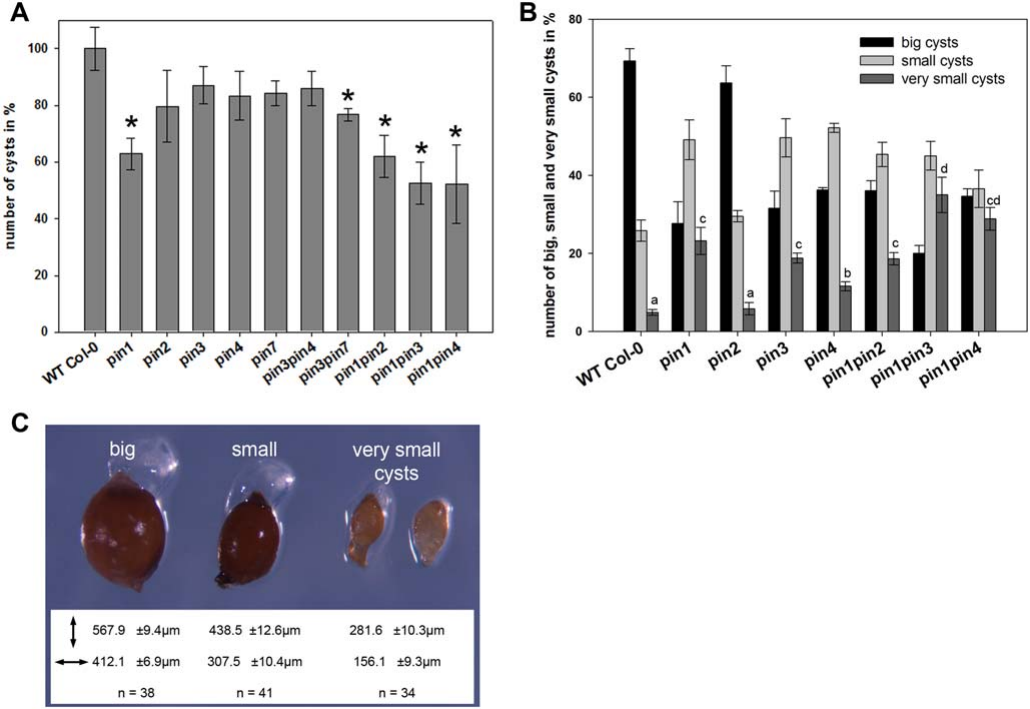
Infection studies of *pin* mutants using the cyst nematode *H. schachtii*. (A) Number of cysts in percentage obtained from several *pin* mutants six weeks after inoculation. Asterisks point to significant values (p<0.05). (B) Number of cysts in percentage obtained from several *pin* mutants six weeks after inoculation and divided in different categories. For the “very small cysts” category, the mutants were statiscally grouped in several classes (a–d; p<0.05). (C) Different categories of cysts obtained from infection experiments on *pin* mutants. Both length and width of the cysts were measured. n = number of cysts measured.

Our findings on specific changes in auxin distribution and modulation of *PIN* expression and localisation during nematode infection indicate that nematodes modulate PIN-dependent auxin transport to manipulate auxin distribution at the feeding site. Indeed, it has been shown previously that activity of different PIN proteins is required for asymmetric auxin distribution in course of many developmental processes [26]. We therefore examined whether *pin* mutations would affect the ability of nematodes to initiate and develop their feeding sites. In three independent infection experiments *pin2*, *pin3*, *pin4*, and *pin7* mutants showed a 10 to 25% reduction in number of cysts (Figure 3A). Interestingly although the *pin3* and *pin4* mutants only resulted in a slightly lower number of cysts, a much higher percentage of small cysts was observed (Figure 3B and 3C). This indicates that in these mutants the development of NFS was hampered and thus that PIN3 and PIN4 are required for NFS development rather than initiation. No difference could be observed in the size of the cysts developing on the *pin2* mutant roots (Figure 3C). The *pin1* loss-of-function mutation showed the highest effect on nematode reproduction, as nematode infection of *pin1* mutants was reduced up to 40% (Figure 3A). Since *PIN1* expression is downregulated in NFS, this implicates that auxin transport from the shoot to the root tip is needed for an efficient nematode infection.

It has been shown that defects in single *pin* mutants can be masked by ectopic activity of the remaining *PIN* genes [27],[28]. Therefore, we infected several mutant combinations for *PIN1*, *PIN2*, *PIN3*, *PIN4* and *PIN7*. Interestingly, the *pin1pin2* double mutant could not further reduce nematode infection compared to the *pin1* single mutant (Figure 3A). Also the *pin3* and *pin4* mutations revealed no synergistic effect on nematode development since the *pin3pin4* double mutant was infected with an efficiency comparable to the corresponding single mutants (Figure 3A). In contrast *pin1pin3*, *pin1pin4*, and *pin3pin7* double mutants showed a lower susceptibility towards *H. schachtii* compared to the corresponding single mutants. Moreover also the number of very small cysts (281.6±10.3 µm length, 156.1±9.3 µm width) significantly increased in the *pin1pin3* and *pin1pin4* mutants (Figure 3C). This suggests that the cyst nematode *H. schachtii* requires both the acropetal auxin transport mediated by PIN1 and PIN7 as well as the induced expression of *PIN3* and *PIN4* in NFS.

## Discussion

The initiation of the feeding structures of plant-parasitic nematodes coincides with an accumulation of the plant signalling molecule auxin. Auxin is transported throughout the plant by means of a complex interacting network of influx and efflux carrier systems. The increased local auxin response in NFS can therefore be the result of increased auxin influx and/or an inhibition of auxin efflux. Indeed activation of the AUX1 promoter could be demonstrated during both cyst and root-knot nematode infection [29], thus suggesting that the AUX1-mediated auxin influx is stimulated in young NFS. In this paper we analysed the role of the PIN auxin efflux carriers for formation and development of NFS. We show that the expression of *PIN1* and *PIN7*, which are expressed in the vasculature, is absent from the early NFS. This indicates that the nematodes directly or indirectly downregulate *PIN1* and *PIN7* expression in order to inhibit auxin transport out of the feeding site initial. On the other hand, acropetal auxin transport along the root is needed to deliver auxin to the infection site. Infection studies revealed that particularly PIN1 is at play during this process. Inoculation of *pin1* mutant seedlings with *H. schachtii* resulted in a 40% reduction of cysts compared to wild-type (similarly reported also by Goverse *et al.* [10]), while *pin7* reduced the infection efficiency only with 20%. These data demonstrate the importance of PIN1-mediated auxin transport during NFS establishment.

After initiation, the NFS expands by incorporating the surrounding cells. Using the auxin transport inhibitor NPA it has been proposed that radial expansion of NFS depend on active auxin transport [10]. We found that both PIN3 and PIN4 are highly and specifically expressed in NFS. Moreover, PIN3 localisation in NFS is changed from a basal to a lateral localisation, strongly suggesting that PIN3-mediated auxin transport is responsible for the shift in local auxin activity towards the surrounding cells. In line with these results, *pin3* and *pin4* did not have a significant effect on NFS initiation but severely affected the size of the cysts. The high percentage of very small cysts demonstrated that nematodes have problems in expanding their feeding site.

Regarding PIN2, our expression analysis could not confirm previously published array data [30]. Gene chip data from soybean (*Glycine max*) roots infected with *H. glycines* identified *PIN2* (GmaAffx.47493.1.S1_at) as being upregulated 2 dpi [30]. However our *PIN2::GUS/GFP* expression data are not surprising, since *PIN2* acts totally different from the other *PIN* genes regarding their expression patterns in uninfected plants. In contrast to the other PINs, *PIN2* is neither expressed during embryogenesis [19] nor in mature root cells nor during early lateral root initiation [21]. Moreover, our infection studies and these from Wubben *et al.* [31] reveal only a mild effect of the *pin2* mutation on nematode infection. Together these data argue for a more moderate role of PIN2 during cyst nematode infection than first suggested [10].

Nonetheless, our studies on the expression and polar, subcellular localization of auxin transport components during nematode infection clearly revealed that nematodes are capable of modulating auxin transport and local auxin activity during initiation and development of NFS. It will be of high interest for future studies to elucidate the mechanism, by which nematodes can manipulate the expression and localisation of the PIN proteins. PIN proteins are continuously cycling between the plasma membranes and endosomal compartments [32]. This vesicle transport is enabled by GNOM, a membrane-associated GDP/GTP exchange factor for small G proteins of the ARF class (ARF GEF) [33]. During the very early events of lateral root initiation, a development process with molecular similarity to NFS initiation [34]–[37], Kleine-Vehn *et al.* (2008) could demonstrate a PIN1 polarity switch from the anticlinal to the outer periclinal side of pericycle cells [38]. Moreover it was shown that these dynamic polarity changes depend on the ARF GEF GNOM vesicle-trafficking regulator [38]. Therefore it would be of interest to investigate whether nematodes can directly or indirectly influence this ARF GEF-dependent transcytosis pathway. Most probably auxin, accumulating in the NFS together with the protein cocktail injected by the nematode, will change cell identity of the infected cell. As a consequence vesicle trafficking can be altered. In this context it will also be interesting to analyse whether the localisation of the AUX1 influx carrier is altered during the formation of NFS. Especially because AUX1 is cycling in a GNOM-independent trafficking pathway that is thus distinct from PIN subcellular trafficking [39].

**Figure 4:**
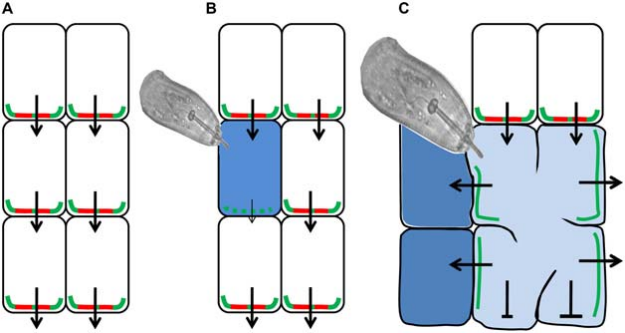
Model of PIN-mediated auxin transport during NFS establishment. (A) Uninfected root cells. (B) In response to nematode infection *PIN1* expression is downregulated in the initial feeding cell, thus hampering PIN1-mediated basipetal auxin transport, and as a result auxin accumulates in this cell. (C) In order to expand their feeding site radially, nematodes direct PIN3 to the lateral cell membranes and consequently auxin is transported laterally towards the surrounding cells. Red lines represent PIN1; green lines represent PIN3; arrows indicate auxin flow; blue colouring illustrates DR5-visualized auxin response.

Using the described results we postulate a model (Figure 4) in which PIN1 (and probably AUX1) is the driving force for NFS initiation. By downregulating *PIN1* in infected pericycle cells, nematodes block the auxin efflux out of these cells (Figure 4B) thereby enhancing the local auxin activity in the infected cells. Reducing the PIN1-mediated auxin transport towards nematode infection sites resulted in a 40% reduction in number of cysts, indicating that almost half of the nematodes capable to infect did not succeed to initiate a NFS. Following initiation, the local auxin activity shifts towards the periphery of the NFS where auxin is presumably needed for the preconditioning of cells prior to their integration into the developing syncytium. PIN3 as well as PIN4 proteins localize to the lateral cell membranes indicating a lateral auxin flow. *H. schachtii* inoculation of the corresponding mutants revealed an increase of very small cysts indicating the presence of underdeveloped NFS. These observations let us hypothesise that PIN3 and PIN4 are needed for the expansion of the feeding site (Figure 4C).

In summary, our study has revealed that cyst nematodes manipulate both the expression and subcellular polar localization of PIN auxin transporters in order to facilitate the initiation and development of their feeding structures and thus infection. An interesting question which can be immediately raised is whether this auxin transport pathway is also recruited by root-knot nematodes. Although giant cells formed by root-knot nematodes differ significantly from cyst nematodes induced syncytia in terms of feeding site morphology, it is very likely that auxin and auxin transport have a similar role in establishing both NFS. For example, a strong and local activation of the DR5 promoter was observed in both young syncytia and giant cells, while 3 to 5 dpi a gradual decrease in DR5 promoter activity could be demonstrated [11]. Also the activation of the AUX1 promoter was similar for both nematode species [29]. It will be a challenge for future studies to elucidate the downstream auxin-dependent pathways activated in NFS in response to the dynamic PIN-dependent auxin gradients induced by both cyst and root-knot nematodes.

## Materials and Methods

### Plant Material and Growth Conditions

PIN1/2/3/4/7-GUS lines were described in [21]; DR5::GUS by [22]; *pin1*, *pin3*, *pin4*, *pin3pin4*, *pin1pin2*, *pin1pin3*, *pin1pin4*, *pin1pin7* by [27], *pin2* by [40], *PIN1::PIN1-GFP* by [21], *PIN3::PIN3-GFP* by Ding *et al.* (submitted). In general plants were grown *in vitro* at 21°C under a 16 h light /8 h dark photoperiod.

### Nematode Culture and Infection Tests

Culture of cyst nematodes (*Heterodera schachtii*) and infection tests using *Arabidopsis thaliana* were done according to [12]. The data were statistically analyzed in SPSS (version 16.0) using an ANOVA-Tukey test (p<0.05).

### Histochemical GUS Staining and Microscopy

Histochemical localization of GUS activity was done according to [12]. GUS stained seedlings were cleared using lactic acid, analyzed using a DIC light microscope (Olympus) and photographed using a Nikon digital camera. The GFP signal of infected *PINx::PINx-GFP* (x stands for 1,2,3,4,7) plants was visualized using a Zeis LSM confocal microscope.

### Accession Numbers

PIN1 (NM_106017.3; NP_177500.1), PIN2 (NM_125091.3; NP_568848.1), PIN3 (NM_105762.2; NP_177250.1), PIN4 (NM_126203.2; NP_565261.1), PIN7 (NM_179369.1; NP_849700.1).

## Supporting Information

Figure S1 Expression pattern of *PIN* genes in uninfected roots. A. *PIN::GUS* activity in the mature main root. B. *PIN::GUS* expression in the primary root tip. C. PIN::PIN-GFP localisation in the primary root tip.
(5.30 MB TIF)

Figure S2 *PIN::PIN-GFP* expression and localisation in NFS of *H. schachtii* at 4 dpi. left, transmission; right, GFP; arrowheads indicate head of nematodes; NFS are outlined.
(2.84 MB TIF)

Figure S3 Time-course experiment on PIN3::PIN3-GFP and PIN4::PIN4-GFP roots infected with *H. schachtii*. (A–F) PIN3::PIN3-GFP infected roots (G–L) PIN4::PIN4-GFP infected roots (A–B, G–H) 12 hpi; (C–D,I–J) 1 dpi; (E–F,K–L) 2 dpi; left, transmission; right, GFP signal; arrowheads indicate the heads of the nematodes.
(2.97 MB TIF)
