# Supplementary figures and images for: Parasitic Nematodes Modulate PIN-Mediated Auxin Transport to Facilitate Infection
Source: PLoS Pathog. 2009 Jan 16;5(1):e1000266. doi: 10.1371/journal.ppat.1000266 (PMC2613529; doi:10.1371/journal.ppat.1000266)

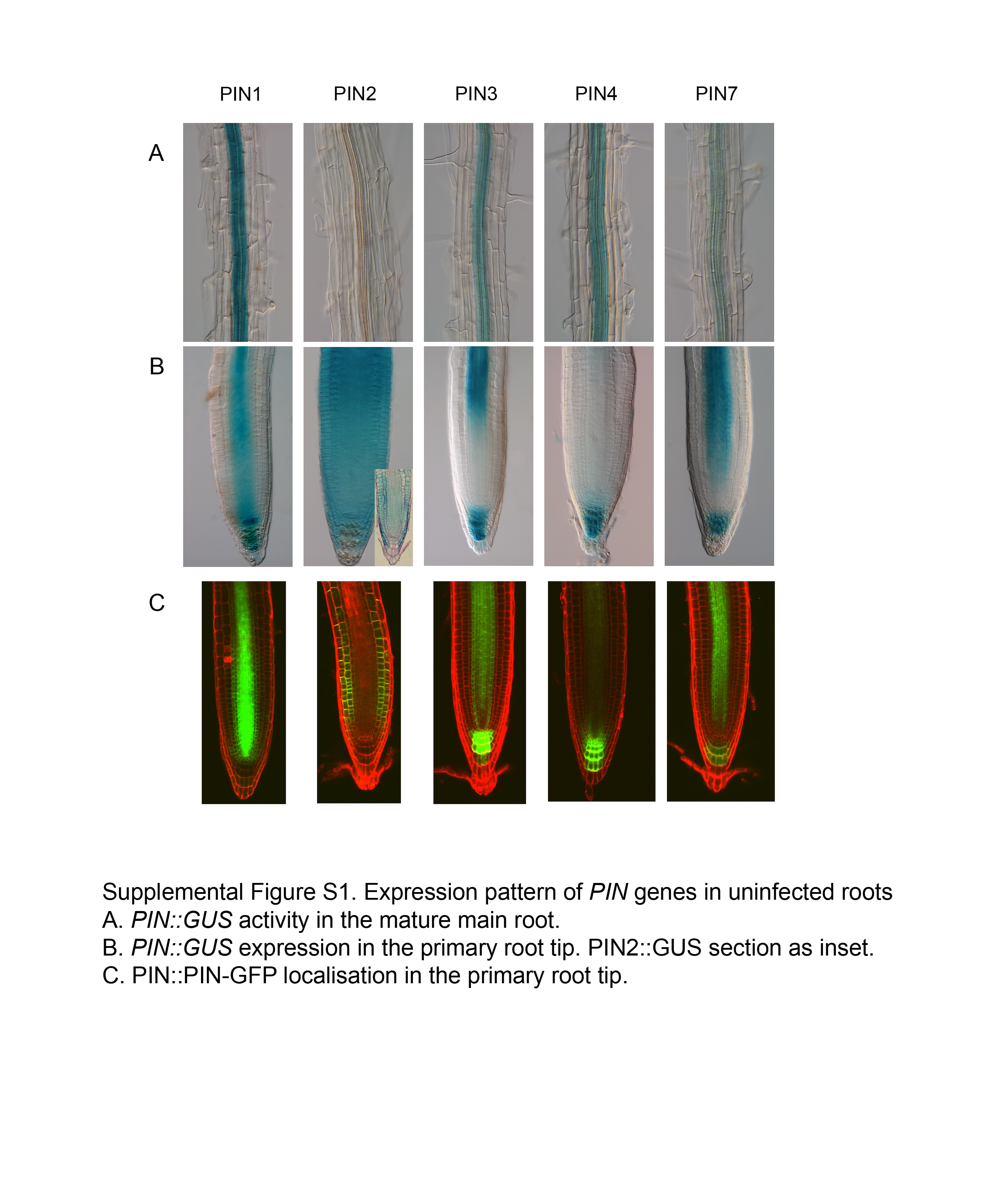

Supplement: Figure S1 — Expression pattern of PIN genes in uninfected roots. A. PIN::GUS activity in the mature main root. B. PIN::GUS expression in the primary root tip. C. PIN::PIN-GFP localisation in the primary root tip. (5.30 MB TIF) [file ppat.1000266.s001.tif]

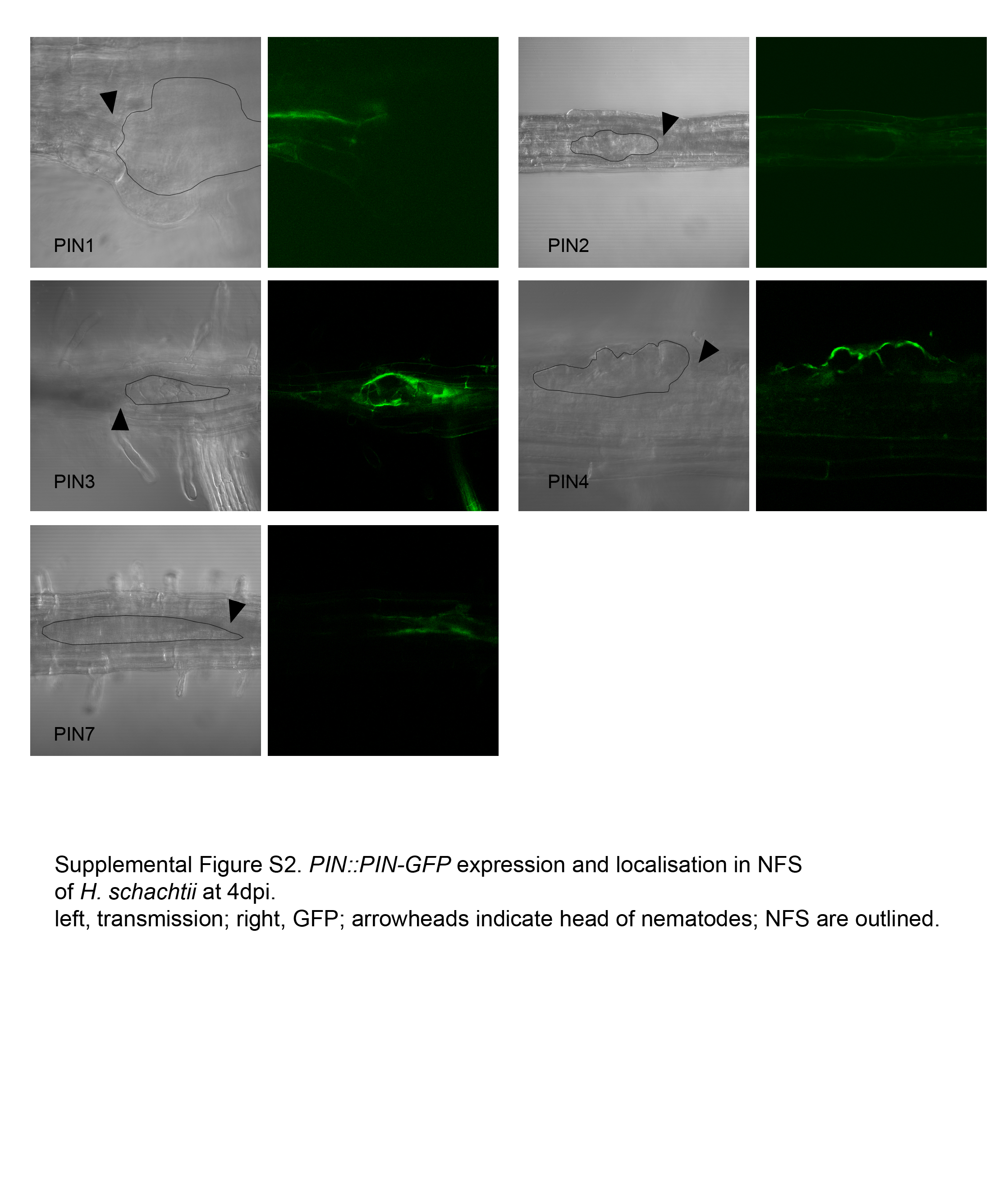

Supplement: Figure S2 — PIN::PIN-GFP expression and localisation in NFS of H. schachtii at 4 dpi. left, transmission; right, GFP; arrowheads indicate head of nematodes; NFS are outlined. (2.84 MB TIF) [file ppat.1000266.s002.tif]

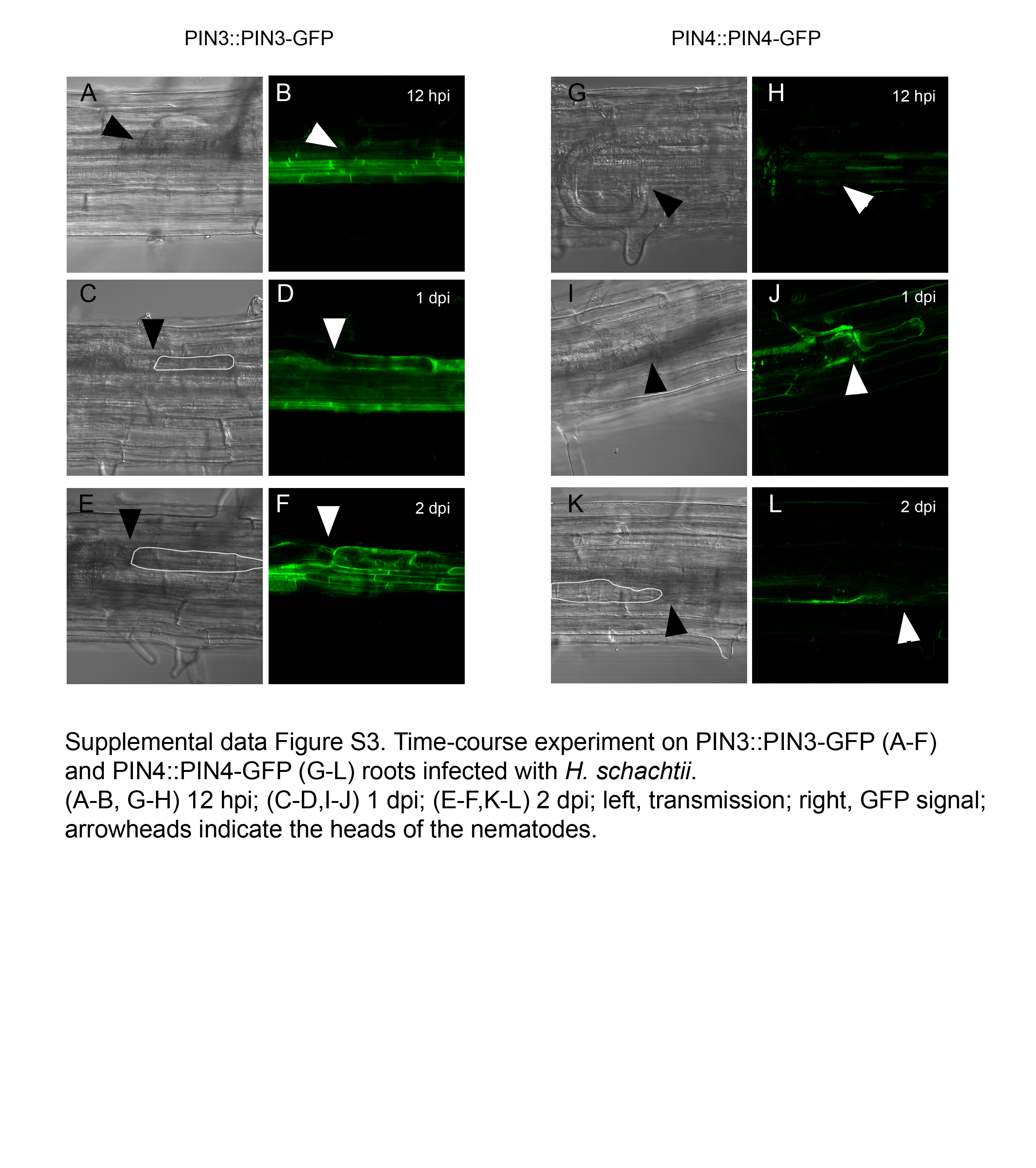

Supplement: Figure S3 — Time-course experiment on PIN3::PIN3-GFP and PIN4::PIN4-GFP roots infected with H. schachtii. (A–F) PIN3::PIN3-GFP infected roots (G–L) PIN4::PIN4-GFP infected roots (A–B, G–H) 12 hpi; (C–D,I–J) 1 dpi; (E–F,K–L) 2 dpi; left, transmission; right, GFP signal; arrowheads indicate the heads of the nematodes. (2.97 MB TIF) [file ppat.1000266.s003.tif]
